# Current status, hotspots and frontiers of ion channel-related research in glioblastoma: a bibliometric analysis from 2005 to 2024

**DOI:** 10.3389/fonc.2025.1588598

**Published:** 2025-06-04

**Authors:** Zefeng He, Jing Liu, Qianxu Jin, Shangkun Ren, Liang Liu, Yingzi Liu

**Affiliations:** Neurosurgery Department, The 4^th^ Hospital of Hebei Medical University, Shijiazhuang, Hebei, China

**Keywords:** glioblastoma, ion channel, apoptosis, bibliometric analysis, tumor microenvironment, single-cell spatial transcriptomics, nanoparticle

## Abstract

Glioma is the most common primary tumor of the central nervous system, with glioblastoma being its predominant pathological type. In recent years, research has demonstrated that ion channels are intricately linked with numerous biological processes in tumor cells, including apoptosis and migration, among others. This study employs bibliometric analysis to systematically examine and synthesize the knowledge structure and research focal points in the field of glioblastoma ion channels. Publications related to ion channels in glioblastoma, published between January 1, 2005 and December 31, 2024, were retrieved from the Web of Science Core Collection (WoSCC). The dataset comprises 764 articles and 234 reviews. Utilize VOSviewer, CiteSpace, Bibliometrix, and Excel to conduct an in-depth analysis and visualization of scholarly contributions based on country, institution, journal, and author. Additionally, examine highly cited publications, references, and keywords. From 2005 to 2024, the number of publications in this field has shown a consistent annual increase. The University of Alabama and Cancer are among the leading institutions and journals. Sontheimer Harald, from the United States, is the most prolific author in this field. The analysis of highly cited publications and co-cited references indicates that the research background and foundation focus on elucidating the mechanisms by which ion channels influence the onset and progression of glioblastoma. Keyword analysis indicated that “tumor microenvironment” (burst strength: 4. 67), “Ca^2+^ activated K^+^ channel” (burst strength: 3. 98), and “chloride channels” (burst strength: 3. 59) have been the keywords exhibiting the highest burst intensity over the past two decades. The keywords that have emerged with higher frequency in the past five years include “tumor microenvironment” (burst strength: 4. 67), “receptor” (burst strength: 3. 11), and “channels” (burst strength: 3. 11). Research on ion channels in glioblastoma has emerged as a prominent and rapidly evolving field of interest. Previous studies have primarily focused on the examination of specific ion channels and their functionalities. However, recent keyword analysis highlights the necessity to explore the interaction between ion channels and the tumor microenvironment. Meanwhile by integrating single-cell spatial transcriptomics and nanoparticle technologies, we can significantly enhance the efficacy of ion channel-targeting therapies.

## 1 Introduction

Glioma is the most common intracranial malignant tumor, comprising 30% of all primary brain tumors and 80% of malignant brain tumors (1). In 2016, the World Health Organization (WHO) revised its classification of central nervous system tumors, categorizing gliomas into low-grade (WHO grades II-III) and high-grade (WHO grade IV) based on their pathological characteristics and malignancy. Low-grade gliomas encompass astrocytoma, oligodendroglioma, and others, while high-grade gliomas primarily include glioblastoma and gliosarcoma (2). Among these, glioblastoma is the most prevalent form. The standard treatment for glioblastoma involves surgical resection followed by adjuvant radiation therapy and chemotherapy. However, due to the development of resistance to cytotoxic agents used in radiation and chemotherapy, glioblastoma has a high likelihood of recurrence (3). Verhoeff et al. investigated the combination of bevacizumab (an inhibitor of vascular endothelial growth factor [VEGF]) with temozolomide for recurrent glioblastoma, but this approach did not improve median overall survival (OS). Imaging studies indicated that antiangiogenic therapy was effective against the permeable regions of the tumor but had limited impact on more aggressive components (4). Feroza Yasinjan’s team highlighted the potential of immunotherapy for glioma treatment, focusing on immune checkpoint inhibitors (ICBs), chimeric antigen receptor (CAR) T cell therapy, vaccine therapy, and oncolytic virus therapy (OV therapy). Despite these promising approaches, no significant therapeutic advancements have been achieved (5). Therefore, there is an urgent need to explore new mechanisms underlying glioblastoma pathogenesis and develop innovative therapies.

As a class of channel proteins, ion channels regulate a selective flux of ions, including sodium (Na^+^), potassium (K^+^), calcium (Ca^2+^), and chloride (Cl^-^) ions, across cell membranes or organelle membranes. Extensive research has established that ion channels play a pivotal role in tumor cell proliferation, migration, apoptosis, and differentiation (6). The impact of ion channels on glioma may be attributed to the activation of relevant signaling pathways (7). Notably, research by Guillaume Jacquemet’s team has demonstrated that L-type voltage-gated Ca^2+^ channels are intimately associated with filopodia formation and stability, thereby promoting glioblastoma cell migration and invasion (8, 9). Consequently, Ca^2+^ channels are significantly implicated in biological behaviors such as glioblastoma proliferation. Glioblastoma cells also express K^+^ and Cl^-^ channels, which are crucial for regulating cell volume and membrane potential. Activation of the Na^+^/K^+^/2Cl^−^ cotransporter facilitates the influx of one Na^+^, one K^+^, and two Cl^-^ ions into the cell, leading to isoosmotic water entry and increased cell volume. Changes in cell size and shape can enhance the aggressiveness of glioblastoma (10). In summary, ion channels represent a critical research direction that can provide valuable insights for developing novel therapeutic strategies for tumors by elucidating mechanisms involved in glioblastoma progression.

Bibliometrics, a branch of scientometrics, involves the quantitative analysis of scholarly outputs using mathematical and statistical methods (11). Through bibliometric analysis, it is possible to conduct an in-depth examination of relevant literature, including analyses of contributions by countries/regions, journals, and authors, as well as high-impact publications, citations, and evolving research hotspots within the field (12). Scientific visualization software is utilized to present this information graphically (13). Although bibliometric studies on glioblastoma exist, no studies specifically focusing on ion channels in glioblastoma have been identified. This study aims to analyze and visualize research on ion channels in the context of glioblastoma from 2005 to 2024, thereby providing insights into the current research landscape and predicting future research trends.

This study leverages data from the Web of Science Core Collection (WoSCC) and employs advanced bibliometric tools such as VOSviewer, CiteSpace, Bibliometrix, and Microsoft Excel to analyze and visualize publications in the field of ion channels in glioblastoma. The analysis focuses on the contributions of countries/regions, institutions, and authors, as well as their collaborative relationships and citation patterns between literatures. Additionally, the evolution of keywords over time is examined. This research reviews the current direction and hotspots in the study of ion channels in glioblastoma and further predicts potential new research directions.

## 2 Materials and methods

### 2.1 Data collection

A comprehensive search of relevant publications from the Web of Science Core Collection database was performed on February 10, 2025. The search focused on glioblastoma ion channels-related publications with final publication dates ranging from January 1, 2005 to December 31, 2024. Initially, 1, 058 relevant publications were identified. Subsequently, 60 non-article and non-review documents were excluded, leaving only articles and reviews in English. Full text files of all selected publications were downloaded in the “Full Record and Cited References” format.

### 2.2 Bibliometric analysis

In this study, the bibliometric analysis was conducted utilizing Biblioshiny (based on R version 4. 3. 1), VOSviewer (version 1. 6. 20), and CiteSpace (version 6. 3. R1).

Launched by Dr. Massimo Aria and Dr. Corrado Cuccurullo, Bibliometrix is an R package that serves as a comprehensive bibliometric analysis tool (14). VOSviewer is a software application designed to create and explore maps based on network data, employing statistical and mathematical methods to analyze various aspects of the literature, including cooperation, co-occurrence, coupling, and co-citation (14, 15). CiteSpace, developed using Java, is a software tool renowned for its characteristics and influence in the field of information visualization (16).

**Figure 1:**
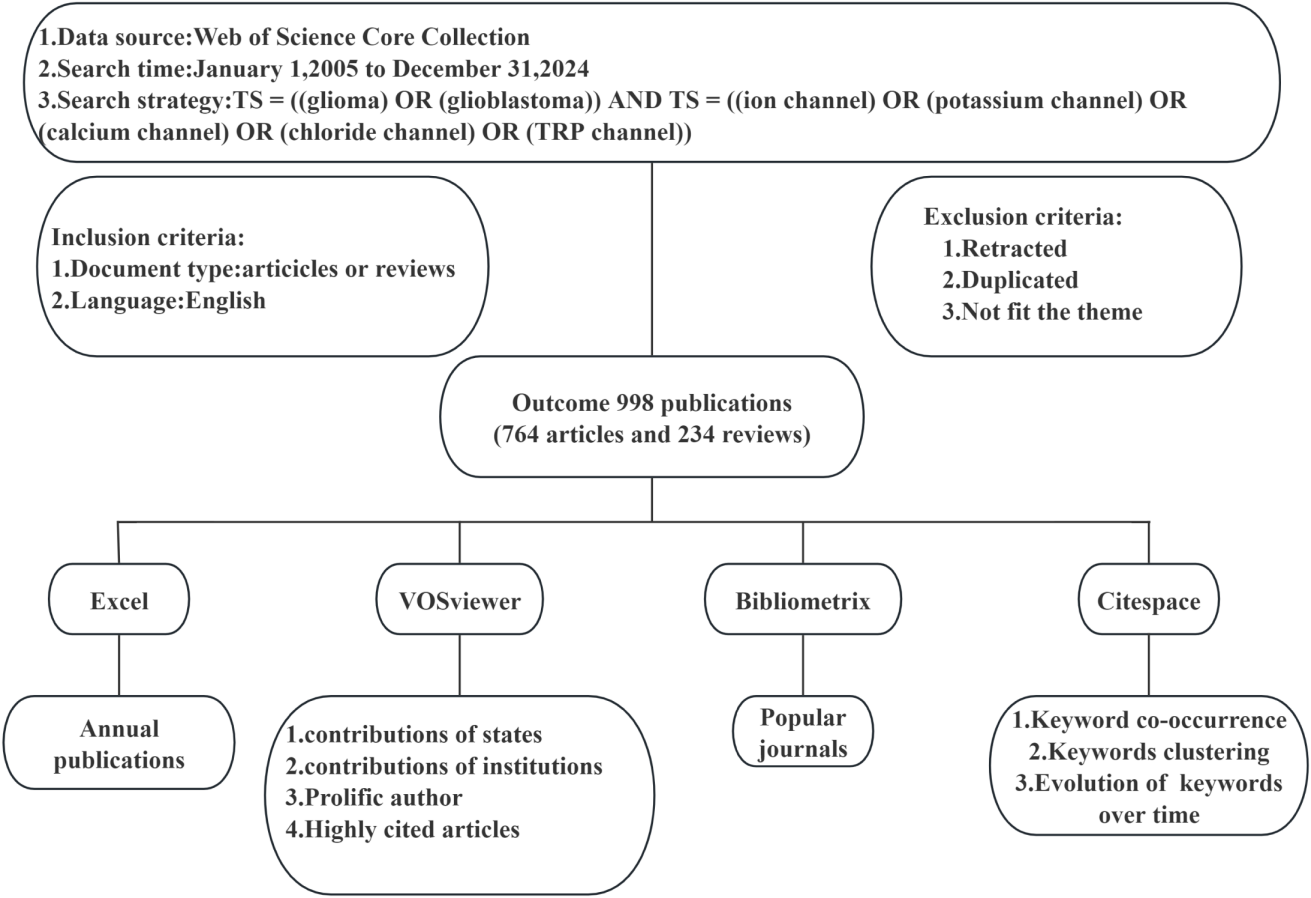
Flowchart of the Retrieval Strategy and Data Analysis Process.

In this paper’s analysis, we initially employed Excel to depict the annual publication trends and classifications, thereby elucidating the evolution of research focus. Using VOSviewer software, we examined the contributions of countries/regions and institutions, generating corresponding world maps and institutional contribution node maps. For authors and journals, we leveraged VOSviewer data to compile tables detailing publication and citation counts. The mapping of prominent journals was conducted using Bibliometrix. Tables for highly cited publications were generated with VOSviewer. Citespace was utilized to analyze citation bursts within references. Regarding keywords, Citespace was used to investigate co-occurrence, clustering, and temporal evolution. **Figure 1** provides a flow chart outlining the search strategy and data analysis process.

## 3 Results

### 3.1 Annual growth trend of publications

**Figure 2:**
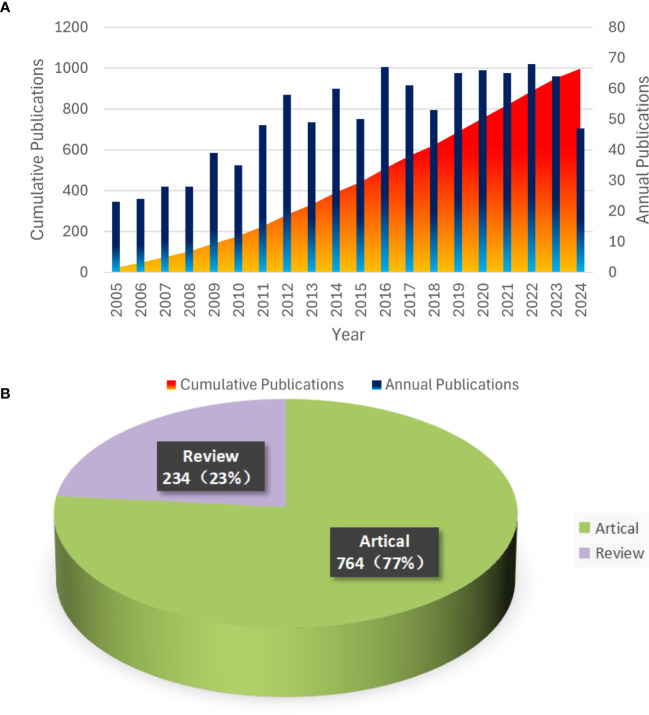
Trends in Annual Publications and Proportions of Article Types from 2005 to 2024. **(A)** Annual Publication Trends and Cumulative Totals. **(B)** Types and Proportions of Publications.

To further investigate the trend of glioblastoma ion channel publications, we utilized Microsoft Excel to visualize the relevant data. **Figure 2A** illustrates the annual publication volume in the field of glioblastoma ion channel research. The bar chart depicts the number of publications per year, which has demonstrated an overall upward trend over the past two decades, indicating that glioblastoma ion channels have progressively become a significant research focus. The cumulative chart displays the accumulated number of publications over time. In 2022, there was an increase of 68 publications, marking the highest annual increment in the past 20 years. Over the past five years, the number of publications has remained consistently high, suggesting rapid development in this field during this period. **Figure 2B** indicates that among all publications in the past 20 years, articles constituted the majority (764), while reviews represented a smaller portion (234).

### 3.2 Major countries/regions and institutions

**Figure 3:**
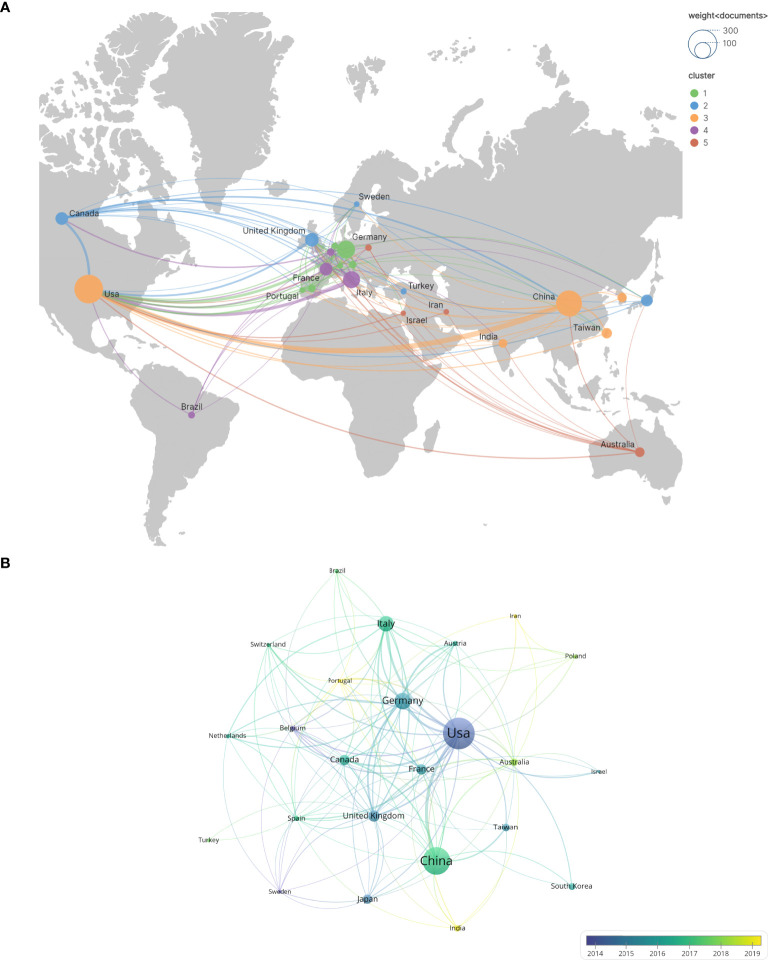
The number of national and regional publications on glioblastoma ion channel-related studies, year of publication, and international collaborations. **(A)** A world map illustrating the distribution of national and regional publications, where differently colored groupings represent similar research directions among collaborating countries. **(B)** Timelines of publications by countries and regions, highlighting trends in publication timelines for each country or region using distinct colors.

In this study, the top 24 countries in terms of publication output were selected, as illustrated in **Figure 3**. The geographical distribution of these countries is depicted in **Figure 3A**. Each node represents a country or region, with the size of the node proportional to its publication count. The links between nodes signify international collaborations, where the thickness of the link reflects the strength of the cooperative relationship. Notably, the United States, China, Germany, Italy, and the United Kingdom are the most prolific contributors. Specifically, the United States leads with 280 publications, followed by China with 266(including Taiwan). Germany, Italy, and the United Kingdom have published 110, 99, and 63 documents, respectively. **Figure 3B** illustrates the temporal trend of national publication counts. Although Portugal, India, and Iran have fewer annual publications, their contributions have primarily emerged in the past five years, indicating their recent engagement in this field. In **Figure 3B**, the United States has established 151 collaborative links, highlighting its extensive international cooperation, whereas China has only 22 links(including Taiwan).

**Figure 4:**
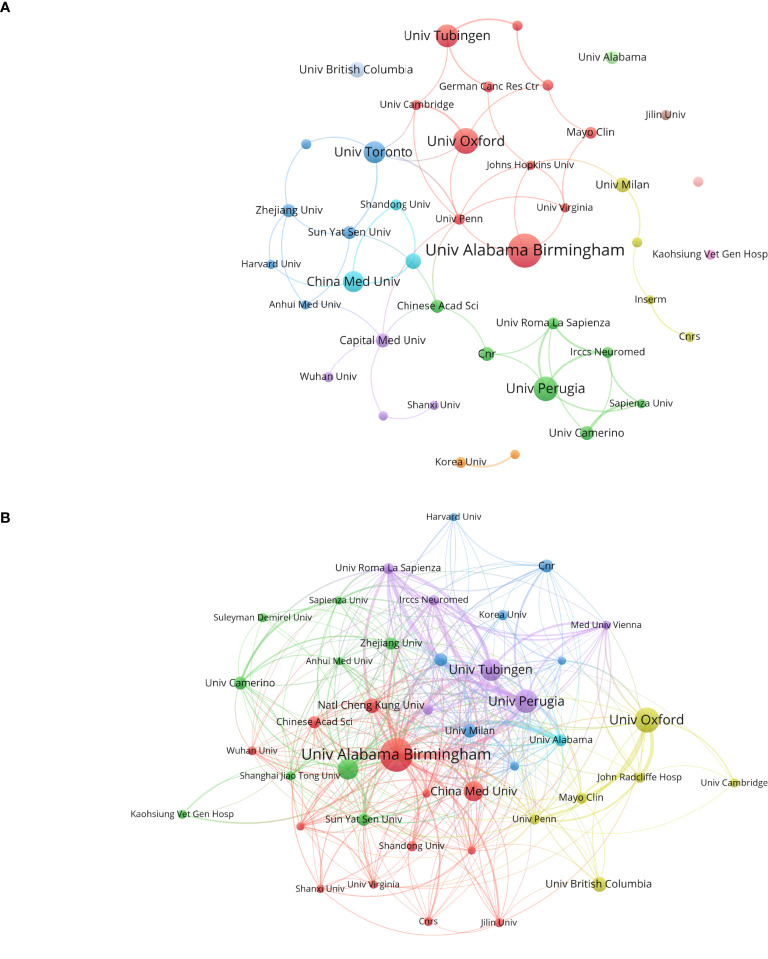
Analysis of research institutions involved in ion channel-related glioblastoma research: This figure presents the number of publications, collaboration patterns, and citation indicators. Color-coded clusters highlight similarities in research topics and directions among institutions. **(A)** Institutional contributions and collaboration networks. **(B)** Inter-institutional cross-reference networks.

**Figure 4** illustrates the publication output and inter-institutional citation relationships within the organization. **Figure 4A** presents the top 42 institutions ranked by publication volume, along with their collaborative partnerships. **Figure 4B** delineates the cross-citation patterns among these leading 42 institutions. Notably, the University of Alabama leads with 37 publications and 3, 348 citations, ranking first in both metrics. This underscores the significant impact and substantial contributions of its research on glioblastoma ion channels to the field. The University of Oxford and the University of Perugia follow closely with 27 and 25 publications, respectively. China Medical University ranks sixth with 21 publications and a total of 6 citation links. It is recommended that Chinese institutions and universities enhance international collaborations, broaden their networks, and increase their influence in this domain.

### 3.3 Analysis of academic journals and evaluation of author contributions

**Figure 5:**
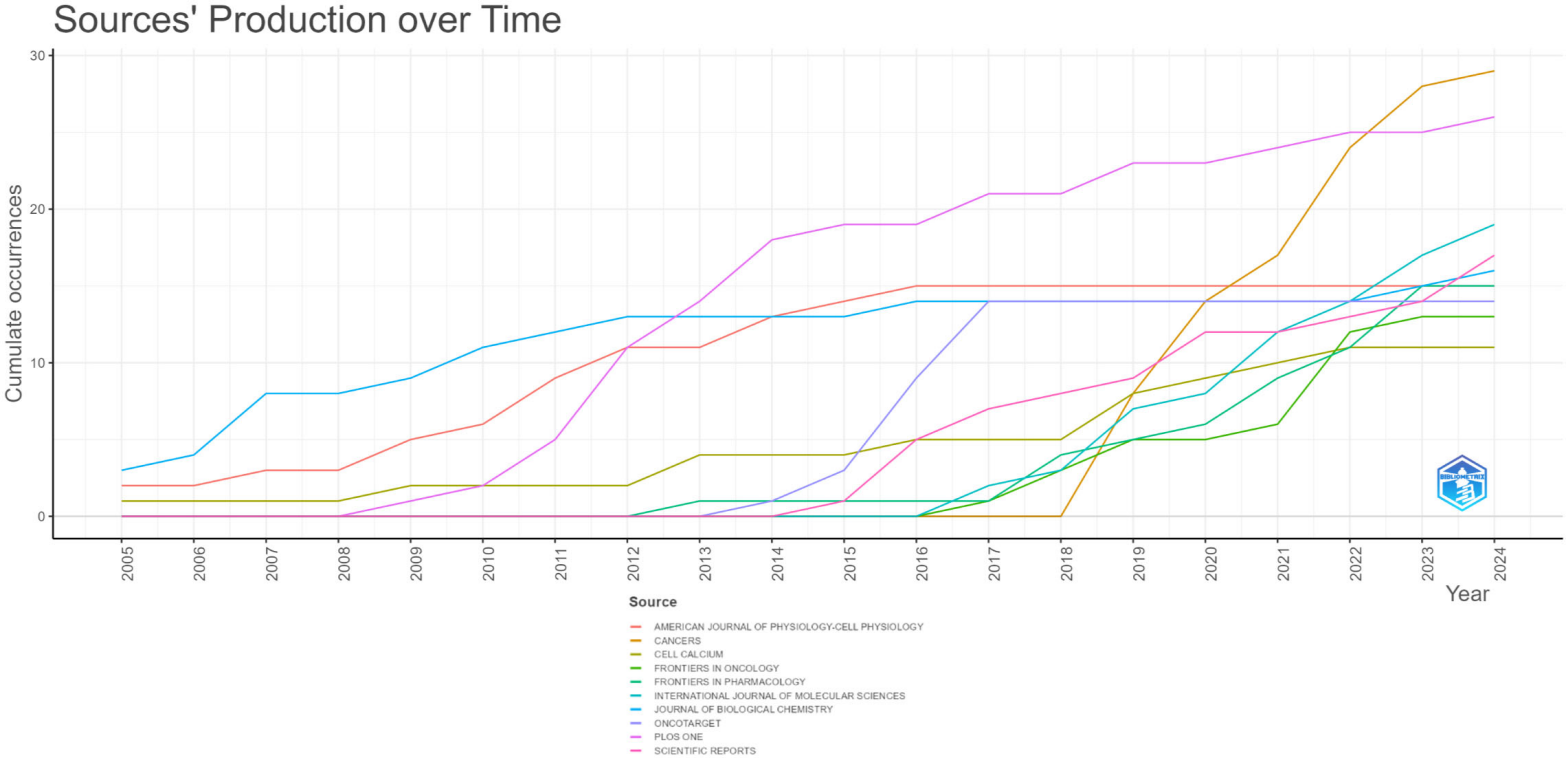
The top 10 journals with the highest cumulative number of published articles over time (each color corresponds to a specific journal).

The top 10 most frequently published journals and their citation frequencies are presented in **Supplementary Table 1**. Among these, *Cancers* has the highest number of published literatures with 29 entries. Following *Cancers* are *PLoS ONE*, *International Journal of Molecular Sciences*, *Scientific Reports*, and *Journal of Biological Chemistry*, with 26, 19, 17, and 16 literatures respectively. Although *PLoS ONE* and *Journal of Biological Chemistry* do not lead in the number of published literatures, their citation counts significantly surpass those of the other eight journals (897 and 887 citations respectively), indicating their high authority in this research field. **Figure 5** provides an analysis of the publication years for the top 10 journals by literature count. As shown in the figure, *Cancers* did not publish any literatures prior to 2018 but has made significant contributions since then, suggesting that its literatures represent current hotspots and frontiers in this research area. Conversely, *American Journal of Physiology-Cell Physiology* held a leading position before 2016, but its publication rate has increased only modestly since then.

**Figure 6:**
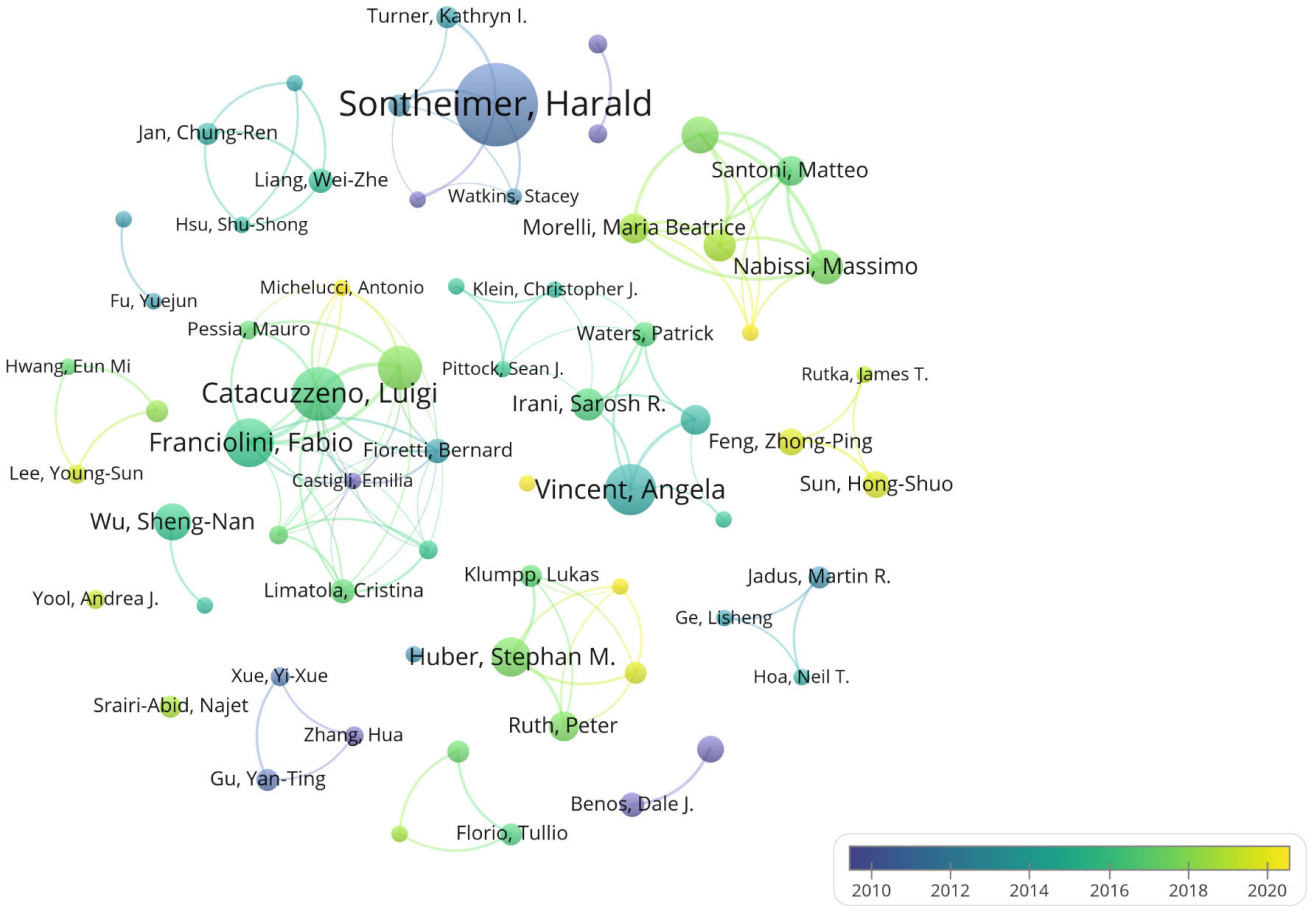
Author collaboration and temporal trends. Different colors indicate the publication trends of various authors over time.

An analysis of author contributions is presented in **Supplementary Table 2**. Sontheimer Harald has made the most significant contribution to this field with 34 publications. Following closely are Catacuzzeno Luigi, Angela Vincent, Fabio Franciolini, and Luigi Sforza, who have published 20, 19, 18, and 16 papers respectively. Notably, three of the top five most prolific authors are from Italy: Fabio Franciolini and Luigi Sforza are affiliated with the University of Perugia. Overall, the analysis of collaboration among authors reveals limited collaborative efforts among researchers, suggesting a need for enhanced cooperation within the field. **Figure 6** illustrates the timeline of authors’ publications and collaborations. It is evident from the chart that all top 10 authors published their literatures before 2020. Zhongping Feng and Hongshuo Sun from the University of Toronto both published six literatures, but their contributions were all post-2020, indicating their significant role in advancing recent research in this field.

### 3.4 Highly cited literatures and co-cited references

In **Supplementary Table 3**, we present the ten most frequently cited literatures that have significantly influenced the direction of this research field and represent the primary focus areas to a considerable extent. The most cited literature is a 2010 publication in BRAIN by Sarosh R. Irani, with 990 citations, which introduced the protein LGI1 (leucine-rich glioma-inactivated protein 1) involved in the formation of voltage-gated potassium channels (VGKC) (17). The second most cited literature, published by Meizan Lai in *LANCET NEUROLOGY*, further elucidates that LGI1, initially isolated from glioblastoma cell lines, is associated with tumor aggressiveness and may function as a potential metastasis suppressor gene (18). The third-ranked literature by Varun Venkataramani suggests that neuromicrotubule-mediated Ca^2+^ signaling between tumor cells contributes to the low cure rate of gliomas (19). These highly cited literatures have extensively analyzed tumor-associated ion channels and explored how these ion channels influence the biological behavior of tumors.

**Figure 7:**
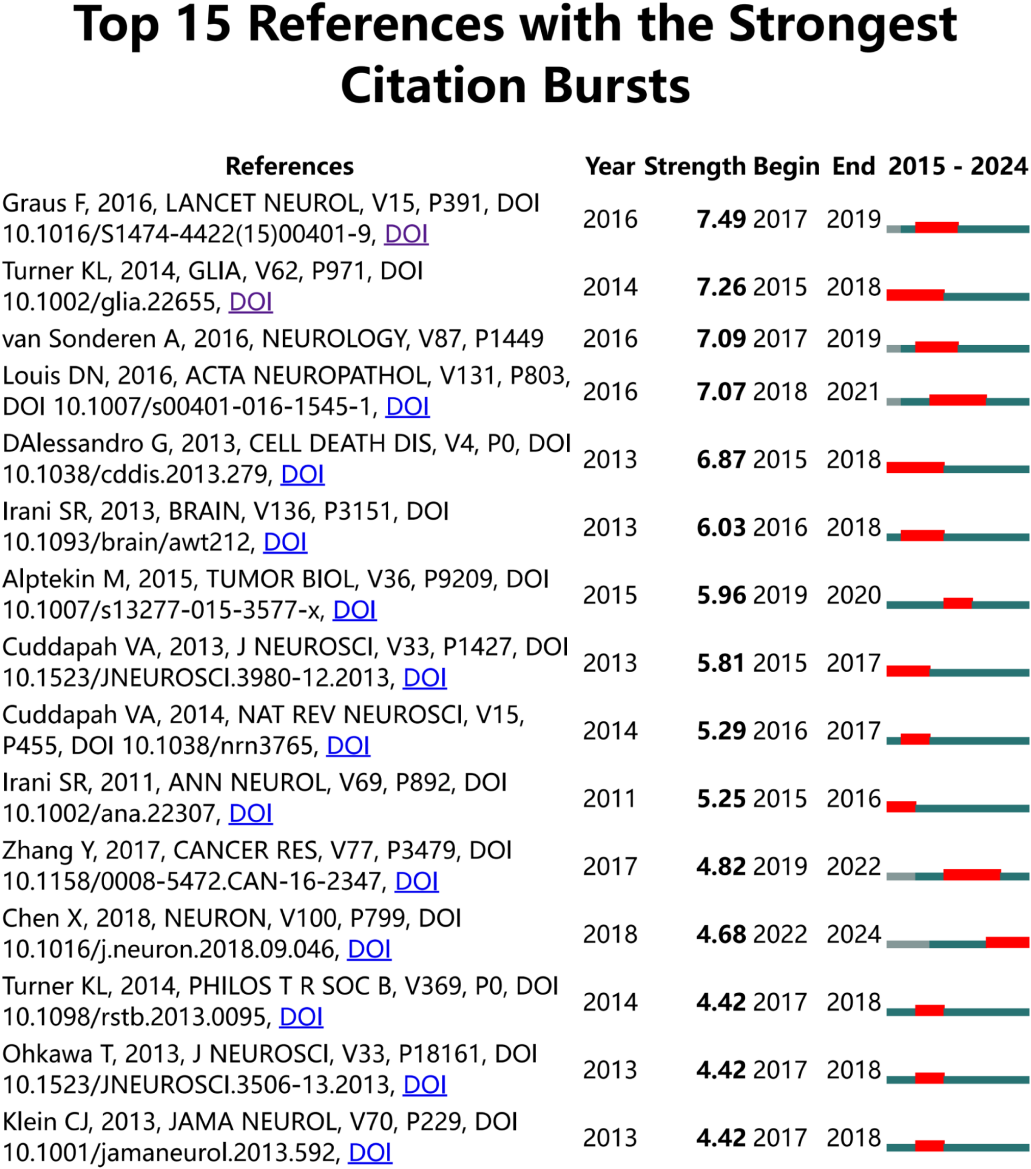
Citation burst analysis of references. The blue line represents the time axis, while the red segments on the time axis indicate the intervals of citation bursts, including the start year, end year, and duration of each burst.

**Figure 7** illustrates the citation burst analysis of cited references. We identified the top 25 most-cited references and ranked them based on their citation intensity. The most prominent reference was a paper by Francesc Graus, published in *Lancet Neurology* in 2016, which discussed leucine-rich glioma-inactivated protein 1 (LGI1), a component of the voltage-gated potassium channel (VGKC) complex (20). The second most cited reference was a study by Kathryn L. Turner in *Glia*, which demonstrated that intracellular Ca^2+^ regulates cell motility and identified the Ca^2+^-activated potassium channel KCa3. 1, overexpressed in 32% of glioma patients. Experimental evidence has shown that KCa3. 1 significantly enhances glioma invasion (21). Among the most frequently cited references, the most recent contribution was made by Xin Chen, who proposed that the interaction between physical forces and signaling pathways can influence tumor development. His research also revealed that PIEZO ion channels, which are mechanically gated cationic channel proteins, promote glioma invasion and proliferation (22).

### 3.5 Co-occurrence, clustering, and hotspot analysis of keywords

**Figure 8:**
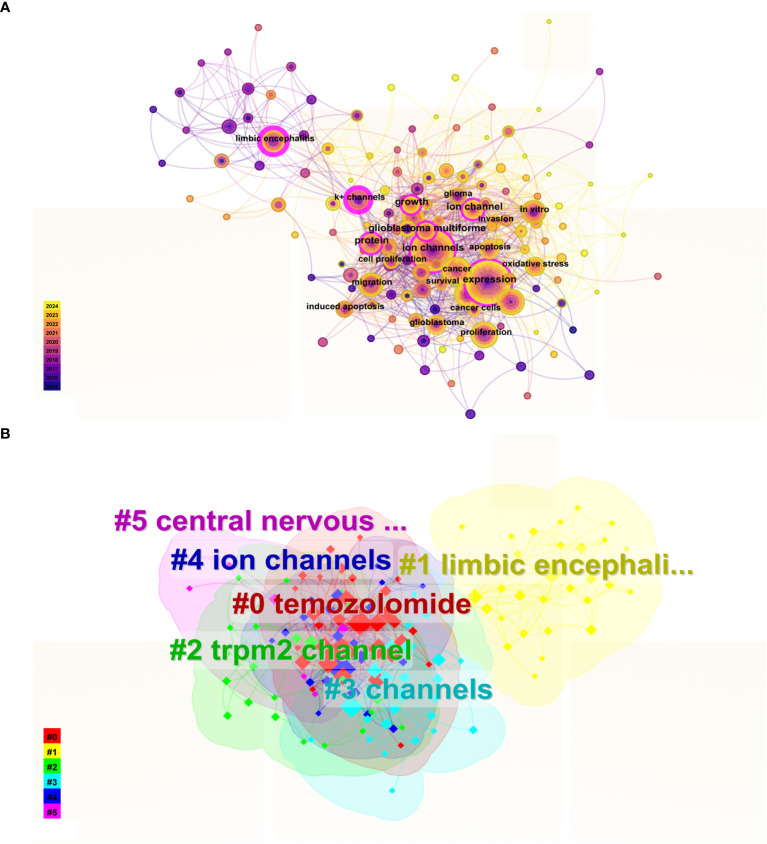
Keyword Co-occurrence and Cluster Analysis. **(A)** In the keyword co-occurrence analysis, nodes represent keywords, lines indicate co-occurrence relationships, and node colors denote the temporal duration of keywords, transitioning gradually from purple (2015) to yellow (2024). Keywords with pink outer rings exhibit higher intermediary centrality, signifying greater influence. **(B)** The keyword cluster analysis illustrates distinct clusters represented by different colors.

The co-occurrence and clustering of keywords are analyzed in **Figure 8**. In the keyword co-occurrence analysis presented in **Figure 8A**, the size of each node reflects the frequency of keyword occurrence, while the lines connecting the nodes indicate the co-occurrence relationships between keywords. The top ten most frequent keywords are “expression, “ “ion channel, “ “cancer, “ “activation, “ “proliferation, “ “inhibition, “ “limbic encephalitis, “ “migration, “ “glioblastoma, “ and “protein. “ In **Figure 8B**, the keywords are clustered into six distinct categories: Cluster 0 (“temozolomide”), Cluster 1 (“limbic encephalitis”), Cluster 2 (“TRPM2 channel”), Cluster 3 (“channels”), Cluster 4 (“ion channels”), and Cluster 5 (“central nervous system”).

**Figure 9:**
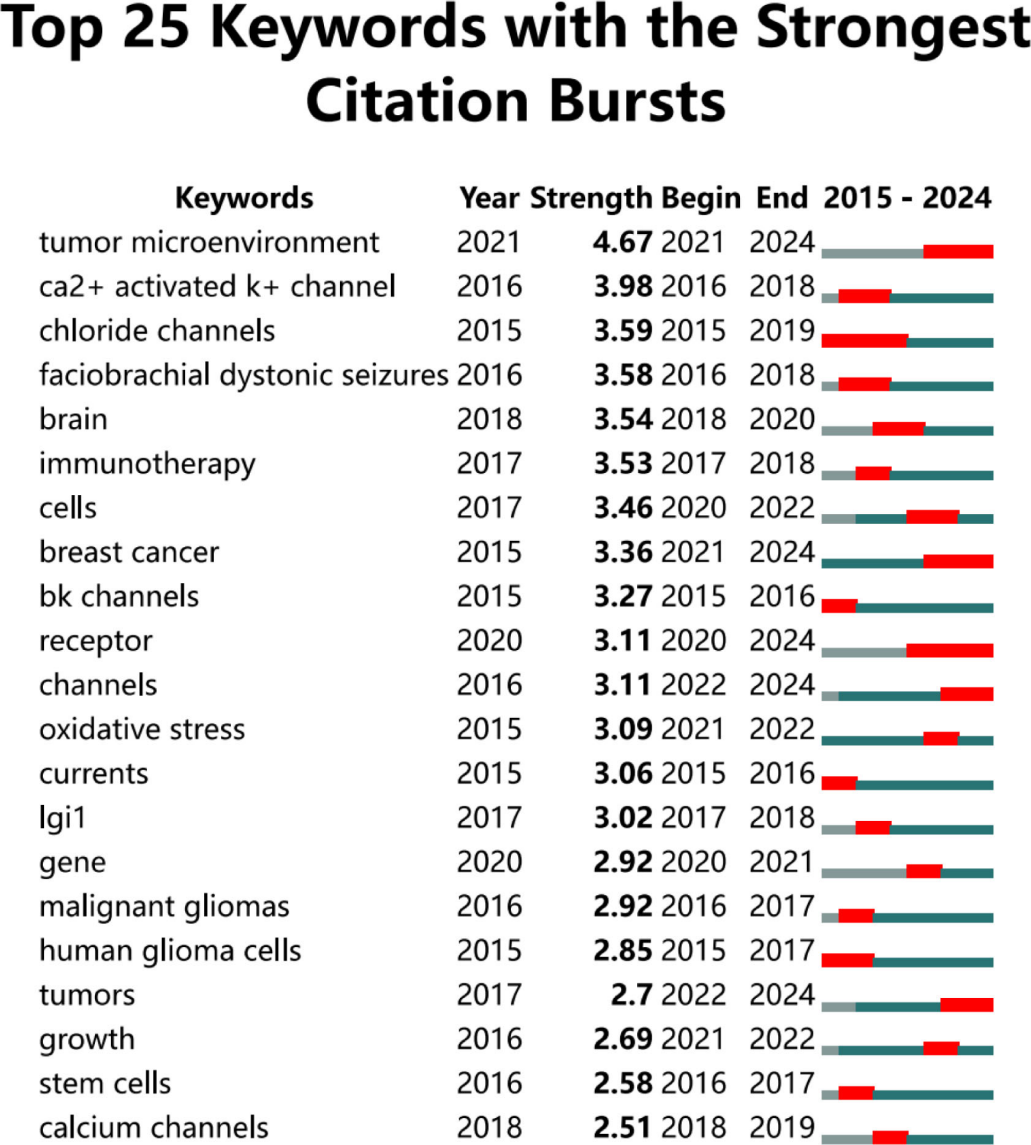
This figure illustrates the top 25 keywords that exhibit the highest citation burst rates.The blue line represents the time axis, while the red segments on the time axis indicate the intervals of keywords bursts, including the start year, end year, and duration of each burst.

We utilized Citespace to identify the 25 keywords with the highest citation burst strength, as illustrated in **Figure 9**, to elucidate the temporal evolution of keyword trends and delineate the research frontier in this field. Over the past two decades, the keywords “tumor microenvironment” (burst strength: 4. 67), “Ca^2+^ activated K^+^ channel” (burst strength: 3. 98), and “chloride channels” (burst strength: 3. 59) exhibited the highest burst intensities. In recent years, particularly within the last five years, the keywords “tumor microenvironment” (burst strength: 4. 67), “receptor” (burst strength: 3. 11), and “channels” (burst strength: 3. 11) have appeared more frequently.

**Figure 10:**
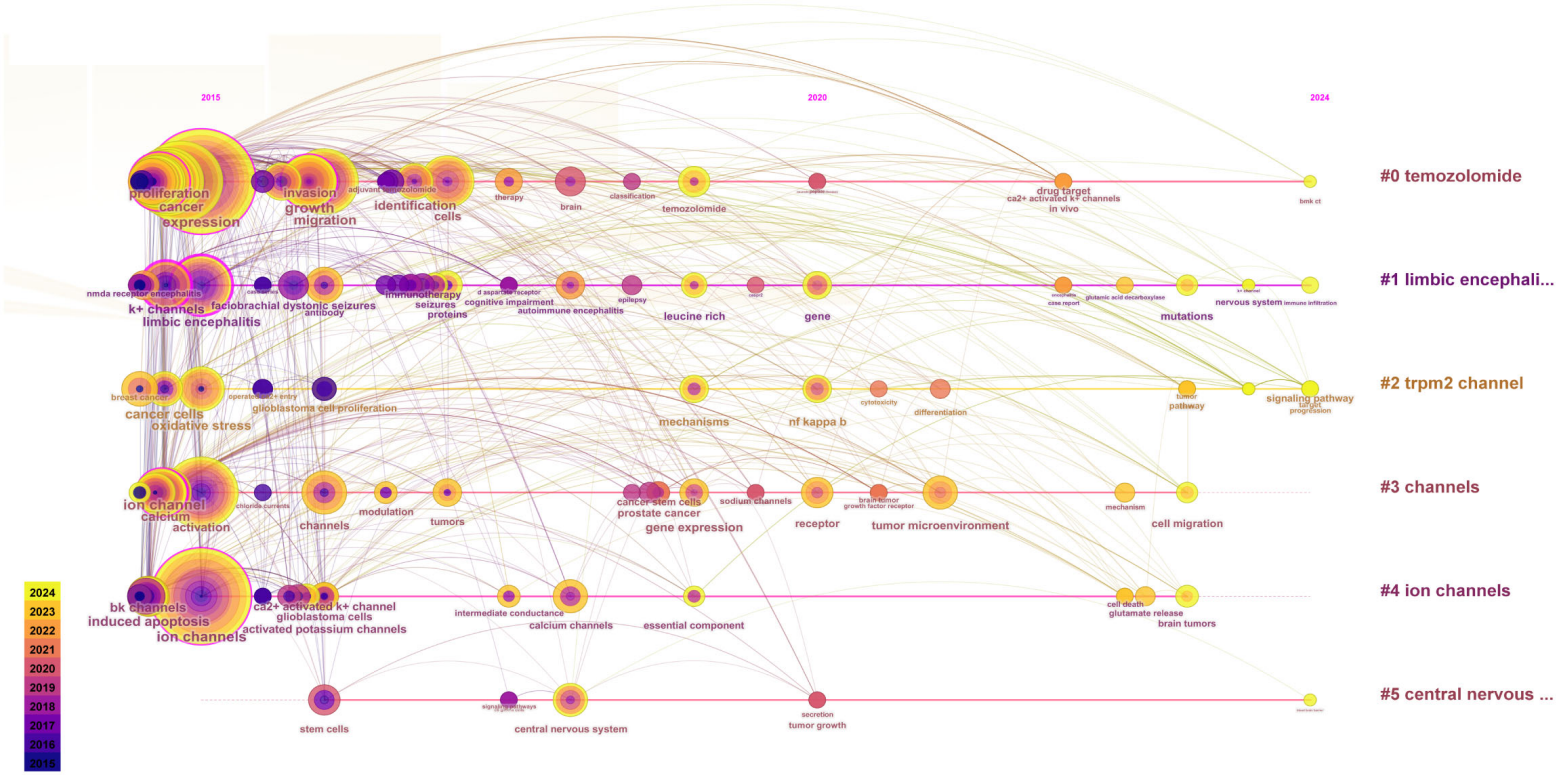
Time evolution of keywords. The horizontal axes, depicted in various colors, correspond to distinct clusters. The position of each node along the horizontal axis denotes the initial appearance time of the associated keyword, while the size of the node is positively correlated with the frequency of the keyword’s occurrence. Lines connecting the nodes indicate co-occurrence relationships. The color gradient reflects temporal proximity: yellower tones signify closer alignment with 2024, whereas purplish hues suggest greater proximity to 2015.

To investigate the evolution and research trends of keywords, we conducted a temporal analysis of keyword occurrences. As illustrated in **Figure 10**, the terms “temozolomide” (Cluster 0), “limbic encephalitis” (Cluster 1), “TRPM2 channel” (Cluster 2), “channels” (Cluster 3), and “ion channels” (Cluster 4) emerged early in the literature. However, only “temozolomide” (Cluster 0), “limbic encephalitis” (Cluster 1), and “TRPM2 channel” (Cluster 2) maintained sustained interest. The term “central nervous system” (Cluster 5) appeared later than the other clusters but gained prominence over time.

## 4 Discussion

### 4.1 Current research status of ion channels in glioblastoma

Glioblastoma is the most aggressive primary malignant brain tumor, characterized by an extremely poor prognosis. This poor prognosis is attributed to several factors, including inadequate delivery of chemotherapy drugs across the blood-brain barrier, intra- and inter-tumor heterogeneity, activation of associated signaling pathways, and the presence of an immunosuppressive microenvironment (23). To further elucidate the mechanisms underlying glioblastoma’s development and progression, our research has identified ion channels as a promising area of investigation. Recent studies in this emerging field have demonstrated that targeting transmembrane ion transporters can effectively modulate tumor behavior and address tumor-related epilepsy (24, 25). We conducted a comprehensive analysis of the literature on glioblastoma ion channels published between 2005 and 2024. Our review focuses on trends in annual publication volumes, national contributions, institutional and author participation, citation patterns, and evolving keyword usage, providing insights into the development and maturation of this research domain.

Over the past two decades, research on ion channels associated with glioblastoma has witnessed substantial progress. The volume of publications in this field has shown a marked increase, rising from 23 literatures in 2005 to 998 literatures in 2024. This rapid growth, particularly from 2020 to 2024, can be attributed to advancements in single-cell sequencing and optogenetics technologies, highlighting potential breakthroughs and emerging research hotspots over the past five years. In terms of country distribution, the United States and China have made significant contributions, ranking first and second with 280 and 266 papers, respectively. The leading position of the United States underscores its advanced experimental facilities and robust financial support. An analysis of frequently cited literature reveals that China’s research focuses on factors influencing tumor-related biological behaviors. Specifically, studies based on classical apoptosis-regulating proteins Bcl-2 and Bcl-xL aim to elucidate their mechanisms of regulating apoptosis, potentially through the formation of ionic conductive channels in synthetic lipid membranes by Bcl-2 and Bcl-xL (26–28), which subsequently modulate tumor cell apoptosis by altering intracellular Ca2+ concentrations (29). At the same time, researchers in the United States are investigating the constituent proteins of specific ion channels and striving to analyze their effects on tumor development. For instance, LGI1 (leucine-rich glioma inactivated protein 1), a component protein of the voltage-gated potassium channel (VGKC), plays a regulatory role in influencing the aggressiveness of glioblastoma (17, 18). Germany’s research focus is on the transmission of information between tumor cells, specifically by investigating the role of synapses and associated ion channels in tumors, followed by an analysis of the factors influencing tumor aggressiveness (19). In the context of international collaboration, the United States leads with 151 partnerships, and its publication count also holds the top position. This highlights the significance of collaborative efforts among nations. In contrast, China has 22 partnerships(including Taiwan), indicating the potential for further enhancement of international cooperation.

The three most prolific authors in this field are Harald Sontheimer from the University of Heidelberg in Germany, Luigi Catacuzzeno from the University of Perugia in Italy, and Angela Vincent from the University of Oxford in the UK. Harald Sontheimer primarily investigated the impact of glutamate on ion channels, which subsequently alters glioma invasiveness and promotes tumor growth. His research indicates that glutamate, as the primary neurotransmitter in the brain, is released at an alarming rate in gliomas (30). Upon release from the presynaptic terminal, glutamate binds to NMDA and AMPA receptors, further promoting Ca^2+^ influx through ion channels. While a high Ca^2+^ environment in normal brain cells leads to cell death, it enhances the invasive capability of tumor cells. Similarly, glioma cells possess Cl^-^ channels on their surface; the efflux of Cl^-^ causes tumor cell shrinkage, thereby promoting tumor invasion in the brain (31–33). Consequently, he proposed salazopyridine, a drug used for chronic inflammatory diseases, which acts on the cystine-glutamate antiporter (Xc^-^) to reduce glutamate secretion and inhibit glioma progression (34). Luigi Catacuzzeno focused on Ca^2+^-activated potassium channels (KCa). Glioblastoma cells regulate intracellular K^+^ and Cl^-^ concentrations via KCa channels to control cell volume, thereby influencing tumor aggressiveness. Additionally, KCa channels can induce “Ca^2+^ oscillations, “ promoting glioblastoma migration (Ca^2+^ oscillations refer to periodic and rhythmic fluctuations in intracellular Ca^2+^ concentration, transmitting various regulatory signals) (35–38). Vincent Angela from the University of Oxford investigates antibodies that target self-ion channels responsible for autoimmune encephalopathy. The voltage-gated potassium channel complex (VGKC complex), a major protein expressed on neuronal surfaces, primarily consists of leucine-rich glioma-inactivated protein 1 (LGI1) and contactin-associated protein 2 (CASPR2). Antibodies against these proteins can lead to autoimmune encephalitis (39, 40). In another study, Vincent suggested that LGI1 might be associated with symptoms of acquired neuromyotonia (41). Regarding the LGI1 protein, previous research has demonstrated its inhibitory effects on the formation and progression of glioblastoma, although it requires ligands to exhibit biological activity (42). By analyzing collaborations among the top 10 authors based on publication frequency, we found that Catacuzzeno and Luigi had the highest collaboration intensity, albeit only 63 instances, indicating a need for enhanced collaboration and communication among researchers in this field.

### 4.2 Highly cited publications and pertinent high-impact references in glioblastoma research

Publications with high citation frequencies frequently signify considerable influence within a given field. The cited references not only reflect current research trends but also provide critical context for the studies, both of which constitute the foundation for further research in this area.

Among the top 10 highly cited publications, Sarosh R. Irani and Meizan Lai have made significant contributions to the field of autoimmune encephalopathy. Specifically, they elucidated the role of leucine-rich glioma-inactivated protein 1 (LGI1) in this condition. Meizan Lai’s research indicated that LGI1 may inhibit glioma metastasis (17, 18), while Sarosh R. Irani focused on treatment and management strategies for autoimmune encephalopathy (43). The remaining seven highly cited literatures primarily addressed ion channel-related studies. Vishnu Anand Cuddapah’s literature provided a comprehensive overview of factors influencing the migration of malignant gliomas, emphasizing that changes in glioma cell permeability affect cell volume and, consequently, migration characteristics. Key mechanisms highlighted include glutamate-mediated increases in Ca^2+^ concentration promoting cell motility, the critical role of Ca^2+^-activated K^+^ channels in migration, tumor cells repurposing NKCC1 and CLC channels to regulate migration, and ligands enhancing migration through Ca^2+^-dependent activation of ion channels. This research offers novel insights into potential targeted therapies for glioma (44). Varun Venkataramani, Natalia Prevarskaya, Feifan Zhou, and Silvia Penuela have all demonstrated a profound interest in the impact of Ca^2+^ concentration on glioma development and invasion. In his research, Varun Venkataramani elucidated the concept of neural microtubule structure. Specifically, in highly malignant glioma cells, synapses are extensively distributed within neural microtubules. AMPAR receptors (a type of ionic glutamate receptor) located at these synapses respond to glutamate stimulation, leading to the opening of Ca^2+^ channels. This process facilitates glioma invasion and proliferation (45). Natalia Prevarskaya’s literature provides an exhaustive overview of the intracellular Ca^2+^ accumulation pathway and the role of associated ion channels. Her research indicates that the TRPM7 channel specifically enhances M-calpain activity by modulating intracellular Ca^2+^ levels. M-calpain can mediate the disassembly of peripheral adhesion complexes or regulate cell adhesion, thereby promoting tumor cell migration. Similarly, TRPM8, which is found in glioblastoma cells, plays an analogous role. Additionally, GPCRs on the surface of glioblastoma cells are stimulated to produce IP3, which acts as an agonist for the IP3R3 receptor, triggering Ca^2+^ release and further promoting tumor cell development and migration (46). In her study, Feifan Zhou investigated the roles of two anti-apoptotic proteins, Bcl-2 and Bcl-xL. These proteins are capable of forming ionic conductive channels in lipid membranes, thereby reducing intracellular Ca^2+^ concentrations to inhibit apoptosis. In glioma cells, overexpression of Bcl-2 has been shown to suppress autophagy mediated by the Beclin1 and Akt-mTOR pathways (47). Silvia Penuela elucidated the concept of panconnexins, detailing their biochemical characteristics and functions. Panx1, a member of the panconnexin family, functions as part of the P2Y receptor (a G-protein-coupled receptor activated by extracellular nucleotides). Upon ATP stimulation, Panx1 triggers the production of IP3, leading to the opening of endoplasmic reticulum Ca^2+^ channels (48). Mathew Tantama focused on analyzing the role of K^+^ concentration changes and introduced the concept that the ATP/ADP ratio is a critical parameter regulating cellular energy state and influencing numerous metabolic activities. Changes in this ratio can modulate ATP-sensitive potassium channels (K_ATP_), with ATP inhibiting channel opening and ADP promoting it (49). It has been demonstrated that inhibition of K_ATP_ channels leads to suppression of ERK (extracellular signal-regulated kinase) activation, which in turn reduces radiotherapy resistance in glioma cells (50). Through the analysis of highly cited literatures, it becomes evident that previous studies predominantly concentrated on the alterations in intracellular and extracellular ion concentrations induced by channel proteins, which subsequently influenced the biological characteristics of tumor cells. These studies encompassed specific channels such as G protein-coupled receptors (GPCRs), voltage-gated calcium channels (VGCCs), ATP-sensitive potassium channels (K_ATP_), and calcium-activated potassium channels (KCa), as well as specific ions including calcium (Ca^2+^), potassium (K^+^), and chloride (Cl^-^).

For co-cited references, we conducted citation burst analysis and ranked them based on citation intensity. The first and third most cited references detailed autoimmune encephalopathy, encompassing clinical diagnostic methods and pathogenesis (20), and introduced the concept of voltage-gated potassium channels (VGKC). Specifically, VGKC includes leucine-rich glioma inactivated protein 1 (LGI1), which is implicated in glioma development (51). The second most cited reference posits that Ca^2+^ can serve as a regulator to activate downstream signaling pathways impacting tumors, introducing a calcium-activated intermediate-conductance potassium channel, KCa3. 1. Experimental evidence confirms that KCa3. 1 plays a regulatory role in the invasion process of malignant gliomas (21). The top three most cited references were primarily cited in earlier years, reflecting past research foci. More recently cited references indicate current research hotspots. Xin Chen, writing in *Neuron*, discovered that PIEZO1, a mechanosensitive ion channel located in the adhesive patch, regulates integrin-FAK signal transmission, promoting tissue hardening through matrix regulation. However, the hardened tissue microenvironment positively feeds back to PIEZO1 channels, increasing their protein expression and thus enhancing glioma cell invasion (23). M. Alptekin asserts that TRP channels play a crucial role in glioblastoma, categorizing identified TRP channels into six families: TRPA (ankyrin transmembrane protein), TRPV (vanilloid), TRPC (canonical), TRPM (melastatin), TRPP (polycystic), and TRPML (mucolipin). Eight TRP channel proteins, including TRPC1, TRPC6, TRPM2, TRPM3, TRPM7, TRPM8, TRPV1, and TRPV2, were found to exhibit significant expression in glioblastoma. The modulation of intracellular Ca^2+^ levels by these channels plays a crucial role in regulating tumor cell proliferation and apoptosis (52). In her research, Ying Zhang investigated the influence of T-type calcium channels on glioblastoma. Specifically, she demonstrated that Cav3. 2, a subunit of the T-type calcium channel, inhibits glioblastoma growth through suppression of the AKT/mTOR pathway and activation of the BAX-mediated apoptotic pathway (53).

### 4.3 Research foundation and frontier

Keyword co-occurrence analysis, cluster analysis, citation burst analysis, and temporal evolution analysis can provide valuable insights into the attention levels, categorization, emergence times, and novelty of keywords. Based on these analytical methods, we have systematically summarized the current research hotspots and foundational areas, and identified potential new research directions.

#### 4.3.1 Foundation of existing research

Through a comprehensive analysis of the keywords, we identified “expression”, “ion channels”, “cancer”, “activation”, and “proliferation” as the five terms with the highest co-occurrence frequency. This indicates that these keywords represent the predominant research focus in this field, particularly concerning how alterations in ion channel expression and activation modify ion concentrations within tumor cells, thereby influencing their proliferation. Additionally, our analysis revealed that “Ca^2+^ activated K^+^ channels”, “chloride channels”, and “BK channels” were early research hotspots. Both “Ca^2+^ activated K^+^ channels” and “BK channels” refer to calcium-activated potassium channels (KCa), which are tetramers composed of alpha subunits first identified in various human neurons in 1972. Based on their conductance levels, KCa channels can be classified into three primary subfamilies: large-conductance (BK), intermediate-conductance (IK), and small-conductance (SK). The activation of KCa channels has been shown to be closely associated with glioblastoma proliferation (54). Previous studies have indicated that KCa channels may contribute to drug resistance in glioblastoma, with significant upregulation observed in mesenchymal glioblastoma. Katrin Ganser et al. demonstrated through *in vitro* experiments that targeting KCa channels could reduce the malignancy of mesenchymal glioblastoma, inhibit tumor spread, and enhance drug therapy sensitivity (55). Furthermore, Elena Dale explored the potential of KCa3. 1 as a microglia target, proposing a novel therapeutic approach (56). Chloride channels represent a significant area of focus in early-stage research. Chloride ions (Cl^-^) are ubiquitously present in cell membranes and organelle membranes, where they play crucial roles in regulating ion concentrations, cell volume, and electrical excitability. The chloride channel family primarily comprises the CLIC channel, CFTR channel (cystic fibrosis transmembrane conductance regulator), ligand-gated GABA chloride channel, and glutamate receptor chloride channel (57). In glioblastoma, the CLIC1 channel protein is overexpressed and correlates with lower survival rates. This overexpression can further enhance the proliferation, clonogenicity, and tumorigenic potential of glial stem/progenitor cells (58). Consequently, the combination of Temozolomide with voltage-gated chloride channel blockers has demonstrated notable efficacy in treating glioblastoma (59).

#### 4.3.2 Promising avenues for future research

In addition to elucidating the early research hotspots of glioblastoma ion channels, we can predict future research directions in this field through a comprehensive analysis of keywords. Our analysis elucidates that “tumor microenvironment, “ “receptors, “ and “ion channels” are emerging as key areas of interest. Consequently, it is imperative to investigate the impact of drugs on ion channels to modulate the tumor microenvironment. This encompasses regulating immune checkpoint expression, altering macrophage polarization, optimizing acid-base conditions within the microenvironment, and influencing tumor cell metabolism. Furthermore, specific ion channel families have been identified as promising therapeutic targets, leading to the selection of targeted drugs based on these findings. However, current methods predominantly rely on bioinformatics for target identification, which may compromise the specificity and accuracy of ion channel targeting. Additionally, the presence of the blood-brain barrier (BBB) poses a significant challenge, as it limits the ability of drugs targeting ion channels to achieve effective concentrations in the brain. Based on these insights, we should integrate emerging scientific and technological advancements with existing standards of care. For instance, single-cell spatial transcriptomics can provide deeper insights into ion channel heterogeneity, while nanoparticle delivery systems can enhance drug specificity and efficacy by simultaneously loading channel inhibitors and immune checkpoint inhibitors.

The tumor microenvironment (TME) constitutes a complex ecological system primarily composed of tumor cells and non-tumor cells, including immune cells, endothelial cells, and tumor-associated fibroblasts. These cellular components play pivotal roles in disease progression mechanisms (60). Ion channels, as proteins embedded in cell membranes and organelle membranes, can modulate the TME and influence tumor development. Transient receptor potential (TRP) channels, which are permeable to Ca^2+^, represent promising targets for modifying the TME, particularly in glioma immunotherapy. TRP channels consist of seven subfamilies, with TRPV2 being a key member that significantly impacts glioma cell development. Single-cell analysis has revealed that TRPV2 is specifically expressed in macrophages and exhibits the highest expression levels among all TRP channels. Activating TRPV2 channels enhances macrophage migration towards tumor cells within the glioma microenvironment, thereby inhibiting tumor cell proliferation (61). Probenecidsol, an uricosuric agent, suppresses glioblastoma proliferation by inhibiting pannexin-1 channels in tumor cells (62). Moreover, Probenecidsol can activate TRPV2 channels (63), although further research is required to confirm its efficacy in inhibiting tumor cell proliferation in glioma through this mechanism. Nonetheless, Probenecidsol holds potential as a therapeutic agent for glioma. TRPM8 is also a member of the TRP channel family. Its aberrant expression in glioblastoma significantly influences tumor cell survival and radioresistance, potentially through interference with the cell cycle via CaMKII, cdc25C, and cdc2 following channel activation (64). Notably, microglia also express TRPM8, and activation of these channels can alter microglial morphology and increase cellular actin levels, thereby promoting microglial migration and positively regulating immune function (65, 66). The dual regulatory role of TRPM8 on both tumor cells and immune cells provides a foundation for targeted drug selection and delivery strategies. VBJ103, a specific inhibitor of TRPM8, reduces pain signaling caused by cold stimulation and alleviates hypothermia by inhibiting TRPM8 activation (67). Consequently, future studies could explore the potential of using VBJ103 to target glioblastoma cells and improve tumor prognosis. Menthol, a natural activator of TRPM8, induces conformational changes in the channel by binding to its transmembrane domain, thereby enhancing TRPM8 expression (68). Studies have shown that menthol application to glioblastoma cells promotes Ca2+ influx and BK channel activation, thus facilitating tumor migration (69). However, research on the use of menthol to target microglia and enhance immune function remains limited, suggesting that future efforts may focus on precise drug delivery, specific immune system activation, and tumor cell elimination. Mechanosensitive ion channels (MIC), an emerging area of research, can influence tumor proliferation by modulating the tumor microenvironment. MICs transduce mechanical stimuli into chemical signals, thereby initiating downstream signaling cascades. Specifically, PIEZO1, a mechanically-gated cation channel protein, regulates the expression of extracellular matrix (ECM) remodeling genes such as TAZ and FHL3, resulting in ECM stiffening, alteration of the tumor microenvironment, and enhancement of tumor malignancy (70). Furthermore, PIEZO1 modulates intracellular Ca^2+^ levels, which subsequently influences the expression of GDF15. As a member of the TGFβ superfamily, GDF15 targets CTLA4, a key immune checkpoint protein, thereby promoting glioma progression (71). GsMTx4, a spider venom peptide isolated from tarantula venom, is the first identified specific inhibitor of mechanosensitive ion channels. It significantly alters intracellular and extracellular Ca^2+^ homeostasis. GsMTx4 inhibits PIEZO1 primarily by reducing mechanical forces on the cell membrane and stabilizing membrane stretch, thus affecting PIEZO1 activation (72). Studies have demonstrated that early administration of GsMTx4 in combination with sonodynamic therapy (SDT) has exhibited significant anti-tumor effects in glioblastoma, potentially mediated by macrophage infiltration (73). However, the efficacy of GsMTx4 as a monotherapy for glioblastoma remains under investigation, requiring further exploration and discovery.

Single-cell spatial transcriptomics integrates the strengths of single-cell RNA sequencing and spatial omics, enabling simultaneous analysis of channel protein expression and spatial localization information, thereby enhancing the precision of therapeutic interventions. In a study by Shijie Hao et al., single-cell RNA sequencing and spatial omics techniques were utilized to analyze the expression of the GRID2 gene in cerebellar Purkinje fibroblasts, which encodes GluD2, a member of the ligand-gated ion channel family (74). Nanoparticles, characterized by their small size, adjustable morphology, and modifiable surface properties, make them ideal carriers for crossing the blood-brain barrier (BBB) (75). Based on this analysis, we identified potential drugs targeting ion channels, including Probenecid, VBJ103, Menthol, and GsMTx4. Notably, indomethacin solid nanoparticles loaded with Menthol can be delivered transdermally, with Menthol enhancing nanoparticle skin absorption (76). However, no studies have demonstrated that indomethacin solid nanoparticles can effectively cross the BBB to exert effects in the brain, indicating a need for further research on the delivery system. Additionally, while studies on nanoparticle delivery of methylene sulfonamide, VBJ103, and GsMTx4 are limited, lipid nanoparticles (LNPs) may offer promising avenues for drug delivery. LNPs exhibit biocompatibility with the BBB, enable controlled coupling of drugs with ligands, and allow for high drug loading concentrations (77). Therefore, developing LNP-based delivery systems loaded with targeted drugs warrants consideration. Polymer nanoparticles exhibit significant advantages in the delivery of drugs and proteins, facilitating targeted delivery and controlled release through optimized surface modifications and internal structures. In glioblastoma therapy, lipid-polymer hybrid nanoparticles have been employed to deliver Cas9 and sgRNA, effectively inhibiting tumor proliferation (78). However, challenges persist in nanoparticle-based delivery systems, including concerns over nanoparticle safety and compensatory activation of ion channels within the tumor microenvironment, which can contribute to therapeutic resistance. To address these issues, a multidisciplinary approach is essential. This includes developing organoid models to simulate drug efficacy and toxicity, integrating comprehensive data to identify optimal targeting strategies, and engineering multifunctional nanoparticles for precise and efficient drug release.

#### 4.3.3 Analysis of clinical translational potential and selection of research models

Through the preceding analysis, it was revealed that the high heterogeneity of glioblastoma is closely associated with its invasiveness and the abnormal expression of specific ion channels within the tumor microenvironment. In recent years, research has increasingly focused on particular ion channels (TRPV2, TRPM8, and PIEZO1), which play a pivotal role in tumorigenesis, drug resistance, and microenvironment remodeling. Consequently, drugs targeting these channel proteins are expected to exhibit promising therapeutic effects and hold significant potential for clinical translation. Studies have demonstrated that TRPV2 is expressed in neural progenitor cells and glioblastoma stem cells (GSCs). Its overexpression has been shown to promote the differentiation of GSCs and reduce the incidence of gliomas both *in vivo* and *in vitro*. In glioblastoma, TRPV2 upregulates the expression of Fas/CD95 and Procaspase-8 mRNA, thereby inhibiting cell proliferation. Furthermore, the activation of TRPV2 enhances the uptake of chemotherapeutic drugs, effectively overcoming tumor drug resistance (79). The drug probenecid, which targets TRPV2, has played a critical role in the treatment of glioblastoma and liver cancer. Zecheng Hu explored the function of TRPV2 in liver cancer cell lines and successfully inhibited tumor formation in a mouse model by using probenecid as an activator of TRPV2 (80). Moreover, related studies have shown that the interaction between propanesulfonate analogs and TRPV2 can offer neuroprotective effects while also providing antiepileptic and anti-inflammatory therapeutic benefits (81). In glioblastoma, probenecid can be combined with α-difluoromethylornithine (DFMO), substantially enhancing DFMO’s anti-tumor activity (82). TRPM8 is significantly upregulated in glioblastoma. Its expression exhibits a negative correlation with patient survival rates and a positive correlation with tumor radioresistance. As a Ca^2+^-permeable cation channel, TRPM8 mediates intracellular Ca^2+^ signaling transduction and interferes with the cell cycle via CamKII, cdc25C, and cdc2, thereby promoting tumor cell proliferation and the development of radioresistance (64). Menthol, a drug that specifically targets TRPM8, enhances Ca^2+^ influx by upregulating TRPM8 expression in glioblastoma, thus augmenting tumor cell migration (83). Furthermore, given that microglia in the brain also express TRPM8, utilizing Menthol to target microglia and promote immune responses within the tumor microenvironment represents an emerging therapeutic strategy. As a member of the Piezo family, PIEZO1 can be exclusively activated by mechanical stimulation. Research has indicated that PIEZO1 is not expressed in normal brain tissue but demonstrates high expression levels in glioblastoma. It primarily promotes tumor proliferation by regulating tumor cell volume and enhancing energy supply to tumor cells. Furthermore, the expression of PIEZO1 upregulates the MMP, MAPK, and PI3K pathways, thereby influencing tumor initiation and progression. In the context of the immune microenvironment, PIEZO1 mediates an increase in intracellular Ca^2+^ levels, which inhibits microglial proliferation and contributes to the establishment of an immunosuppressive microenvironment (84). As an inhibitor of mechanically sensitive ion channels, GsMTx4 enhances the therapeutic efficacy of tumor treatment by specifically targeting PIEZO1. Research conducted by Arianna Buglione has shown that GsMTx4 effectively antagonizes the cellular response to physical mechanical stimuli, thereby suppressing the migration of osteosarcoma cells (85). Meanwhile, to rigorously validate the clinical efficacy of the aforementioned drugs and simulate the interaction between the tumor microenvironment and ion channels, the organoid model technology can be employed. By leveraging advancements in stem cell culture techniques, this approach enables the generation of three-dimensional tissues *in vitro*, which can more accurately recapitulate the structural organization, specific functions, molecular features, genomic alterations, and tumor microenvironment of primary tumors (86). Xin Cui developed a three-dimensional microfluidic angiogenesis model in his research, further investigating the role of cytokines within the immune microenvironment of glioblastoma and their impact on tumor proliferation (87). Consequently, it can be inferred that employing organoid models may provide a more accurate assessment of drug efficacy, thereby enhancing the credibility of the results.

## 5 Strengths and limitations

We provide a thorough analysis of the advancements in glioblastoma ion channels over the past two decades. This study utilizes bibliometric analysis to forecast potential future research hotspots, thereby offering valuable insights for researchers in this field. However, it is important to acknowledge certain limitations in our analysis. Firstly, we were constrained to English-language literature from the Web of Science database, which may have resulted in the exclusion of valuable non-English literature and studies from other databases. Secondly, our inclusion criteria were limited to reviews and articles papers, potentially overlooking conference proceedings and letters that also carry significant academic weight.

## 6 Conclusions

Based on the aforementioned analysis, summarizing national contributions and publication trends, it is evident that research on glioblastoma ion channels has witnessed a significant increase in recent years. The United States has made the most substantial contribution, closely followed by China. The University of Alabama has emerged as the leading institution in this field. Notable contributors include Harald Sontheimer, Luigi Catacuzzeno, and Angela Vincent, among others. The journal *Cancers* has been the most active platform for publishing related studies. Over the past two decades, the primary focus has been on Ca^2+^ channels and Cl^-^ channels. Recently, the interaction between the tumor microenvironment and ion channels has become a prominent area of interest. Additionally, the integration of drug targeting ion channels to modify the microenvironment, coupled with emerging scientific and technological advancements, presents a promising therapeutic approach.

## Glossary

WoSCC: Web of Science Core Collection
WHO: World Health Organization
VEGF: vascular endothelial growth factor
OS: overall survival
ICBs: immune checkpoint inhibitors
CAR: chimeric antigen receptor
OV therapy: oncolytic virus therapy
Na^+^: sodium ions
K^+^: potassium ions
Ca^2+^: calcium ions
Cl^-^: chloride ions
LGI1: leucine-rich glioma-inactivated protein 1
VGKC: voltage-gated potassium channels
KCa: Ca^2+^-activated potassium channels
PIEZO: Piezoelectric
Bcl-2: B-cell lymphoma/leukemia-2
Bcl-xL: B-cell lymphoma-extra lage
NMDA: N-Methyl-D-Aspartate Receptor
AMPA: α-amino-3-hydroxy-5-methyl-4-isoxazole propionic acid
CASPR2: contactin-associated protein 2
TRPM7: Transient Receptor Potential Melastatin 7
TRPM8: Transient Receptor Potential Melastatin 8
GPCRs: G protein-coupled receptors
IP3: Inositol1,4,5-trisphosphate
IP3R3: Inositol1,4,5-trisphosphate Receptor Type3
K_ATP_: ATP-sensitive K^+^ channels
ERK: extracellular signal-regulated kinase
VGCCs: voltage-gated calcium channels
BBB: blood-brain barrier
TME: The tumor microenvironment
TRP: Transient receptor potential
CaMKII: Calcium/Calmodulin-dependent Protein Kinase II
cdc25C: Cell Division Cycle 25 Homolog C
cdc2: Cell Division Cycle 2
MIC: Mechanosensitive ion channels
ECM: expression of extracellular matrix
GDF15: Growth Differentiation Factor 15
TGF: Transforming Growth Factor-beta
CTLA4: Cytotoxic T Lymphocyte-Associated Antigen 4
SDT: sonodynamic therapy
LNPs: lipid nanoparticles
Cas9: CRISPR-associated protein 9
