# Supplementary material for: Current status, hotspots and frontiers of ion channel-related research in glioblastoma: a bibliometric analysis from 2005 to 2024
Source: Front Oncol. 2025 Jun 4;15:1588598. doi: 10.3389/fonc.2025.1588598 (PMC12174454; doi:10.3389/fonc.2025.1588598)
Supplement: Supplementary file 1 [file Table1.docx]

Supplementary table 1.10 Journals with Highest Publication Output from 2005 to 2024.

| **Rank** | **Journal** | **Publications** | **Citations** |
| --- | --- | --- | --- |
| 1 | Cancers | 29 | 521 |
| 2 | PLoS One | 26 | 897 |
| 3 | INTERNATIONAL JOURNAL OF MOLECULAR SCIENCES | 19 | 386 |
| 4 | Scientific Reports | 17 | 345 |
| 5 | JOURNAL OF BIOLOGICAL CHEMISTRY | 16 | 887 |
| 6 | AMERICAN JOURNAL OF PHYSIOLOGY-CELL PHYSIOLOGY | 15 | 703 |
| 7 | Frontiers in Pharmacology | 15 | 261 |
| 8 | Oncotarget | 14 | 689 |
| 9 | Frontiers in Oncology | 13 | 295 |
| 10 | CELL CALCIUM | 11 | 524 |

Supplementary table 2.10 Top Authors Ranked by Publication Output from 2005 to 2024

| **Rank** | **Author** | **Publications** | **Citations** | **Total link strength** |
| --- | --- | --- | --- | --- |
| 1 | Sontheimer, Harald | 34 | 3422 | 24 |
| 2 | Catacuzzeno, Luigi | 20 | 685 | 63 |
| 3 | Vincent, Angela | 19 | 1961 | 22 |
| 4 | Franciolini, Fabio | 18 | 664 | 57 |
| 5 | Sforna, Luigi | 16 | 467 | 49 |
| 6 | Huber, Stephan M. | 14 | 513 | 26 |
| 7 | Santoni, Giorgio | 13 | 493 | 48 |
| 8 | Wu, Sheng-nan | 13 | 150 | 5 |
| 9 | Nabissi, Massimo | 12 | 475 | 47 |
| 10 | Amantini, Consuelo | 11 | 261 | 43 |
| 11 | Irani, Sarosh R. | 11 | 1818 | 17 |

Supplementary table 3. Top 10 most cited literature

| **Rank** | **Documents** | **Year** | **Journal** | **Citations** | **Author** |
| --- | --- | --- | --- | --- | --- |
| 1 | Antibodies to Kv1 potassium channel-complex proteins leucine-rich, glioma inactivated 1 protein and contactin-associated protein-2 in limbic encephalitis, Morvan’s syndrome and acquired neuromyotonia | 2010 | BRAIN | 990 | Sarosh R. Irani |
| 2 | Investigation of LGI1 as the antigen in limbic encephalitis previously attributed to potassium channels: a case series | 2010 | LANCET NEUROLOGY | 751 | [Meizan Lai](https://pubmed.ncbi.nlm.nih.gov/?term=Lai+M&cauthor_id=20580615) |
| 3 | Glutamatergic synaptic input to glioma cells drives brain tumour progression | 2019 | NATURE | 628 | [Varun Venkataramani](https://pubmed.ncbi.nlm.nih.gov/?term=Venkataramani+V&cauthor_id=31534219) |
| 4 | A neurocentric perspective on glioma invasion | 2014 | Nature Reviews Neuroscience | 613 | [Vishnu Anand Cuddapah](https://pubmed.ncbi.nlm.nih.gov/?term=Cuddapah+VA&cauthor_id=24946761) |
| 5 | Calcium in tumour metastasis: new roles for known actors | 2011 | Nature Reviews Cancer | 496 | [Natalia Prevarskaya](https://pubmed.ncbi.nlm.nih.gov/?term=Prevarskaya+N&cauthor_id=21779011) |
| 6 | The CAP Superfamily: Cysteine-Rich Secretory Proteins, Antigen 5, and Pathogenesis-Related 1 Proteins—Roles in Reproduction, Cancer, and Immune Defense | 2008 | ENDOCRINE REVIEWS | 393 | [Gerard M Gibbs](https://pubmed.ncbi.nlm.nih.gov/?term=Gibbs+GM&cauthor_id=18824526) |
| 7 | Bcl-2 and Bcl-xL play important roles in the crosstalk between autophagy and apoptosis | 2010 | FEBS Journal | 355 | [Feifan Zhou](https://pubmed.ncbi.nlm.nih.gov/?term=Zhou+F&cauthor_id=21182587) |
| 8 | Imaging energy status in live cells with a fluorescent biosensor of the intracellular ATP-to-ADP ratio | 2013 | Nature Communications | 329 | [Mathew Tantama](https://pubmed.ncbi.nlm.nih.gov/?term=Tantama+M&cauthor_id=24096541) |
| 9 | Faciobrachial dystonic seizures: the influence of immunotherapy on seizure control and prevention of cognitive impairment in a broadening phenotype | 2013 | BRAIN | 304 | [Sarosh R Irani](https://pubmed.ncbi.nlm.nih.gov/?term=Irani+SR&cauthor_id=24014519) |
| 10 | The biochemistry and function of pannexin channels | 2012 | BIOCHIMICA ET BIOPHYSICA ACTA-BIOMEMBRANES | 292 | [Silvia Penuela](https://pubmed.ncbi.nlm.nih.gov/?term=Penuela+S&cauthor_id=22305965) |
